# Hotspot to Homogeneous: Amorphous Interfacial Current Redistribution Enables Stable Solid-State Lithium-Metal Batteries

**DOI:** 10.1007/s40820-026-02347-w

**Published:** 2026-08-27

**Authors:** Cuiyun Yang, Xupeng Lu, Yexin Pan, Qimeng Zhang, Ruohan Yu, Rongliang Yang, Huan Liu, Molong Duan, Mitch Guijun Li, Ziyi Zhu, Chenghao Yang

**Affiliations:** 1 Division of Integrative Systems and Design, The Hong Kong University of Science and Technology, 999077 Hong Kong SAR, People’s Republic of China; 2 School of Environment and Energy, South China University of Technology, Guangzhou, 510006 People’s Republic of China; 3 The Sanya Science and Education Innovation Park of Wuhan University of Technology, Sanya, 572000 People’s Republic of China; 4 Department of Mechanical Engineering, City University of Hong Kong, Hong Kong SAR, People’s Republic of China; 5 Department of Mechanical and Aerospace Engineering, The Hong Kong University of Science and Technology, 999077 Hong Kong SAR, People’s Republic of China; 6 Department of Electronic and Computer Engineering, The Hong Kong University of Science and Technology, 999077 Hong Kong SAR, People’s Republic of China; 7 Center for Smart Manufacturing, The Hong Kong University of Science and Technology, 999077 Hong Kong SAR, People’s Republic of China; 8 National and Local Joint Engineering Research Center of Lithium-ion Batteries and Materials Preparation Technology, Key Laboratory of Advanced Battery Materials of Yunnan Province, School of Metallurgical and Energy Engineering, Kunming University of Science and Technology, Kunming, 650093 People’s Republic of China

**Keywords:** Solid-state batteries, Electrolytes, Oxide ceramics, Grain boundary hotspots, Laser

## Abstract

Interfacial instability in oxide ceramic electrolyte (OCE)-based solid-state lithium metal batteries (SSLMBs) is conventionally attributed to chemical incompatibility or mechanical failure, yet the underlying atomic-scale mechanisms remain elusive. Here, we reveal that grain boundaries (GBs) in polycrystalline OCEs function as bipolar interfacial hotspots, accelerating three degradation pathways: lowering barriers for Li dendrite nucleation and enabling electron-leakage-driven reduction at anode side, while generating localized overpotentials for cathode phase transformation. To deactivate these GB-driven hotspots, we develop a laser-induced amorphization strategy that constructs a GB-free amorphous interlayer capable of homogenizing Li^+^ flux and blocking electron migration. Applied to a representative sodium superionic conductor-type electrolyte, Li_1.3_Al_0.3_Ti_1.7_(PO_4_)_3_, this approach delivers substantially increased critical current density in Li symmetric cells (1.4 to 2.4 mA cm^−2^) with stable cycling over 2000 h, and achieves an exceptional capacity retention of 101.9 mAh g^−1^ after 800 cycles in LiCoO_2_ full cells operated at 4.5 V. The generality of this strategy is further validated on garnet-type and perovskite-type OCEs. This work introduces amorphous interfacial current redistribution as a universal paradigm for engineering stable interfaces, providing a critical atomic-scale interface engineering route to unlock high-voltage, dendrite-free SSLMBs.

**Supplementary Information:**

The online version contains supplementary material available at https://doi.org/10.1007/s40820-026-02347-w.

## Highlights

- Surface grain boundaries in oxide electrolytes are identified as bipolar interfacial hotspots that actively drive lithium dendrite growth and cathode degradation through electron leakage and current focusing.
- A laser-induced amorphous interlayer eliminates these hotspots via interfacial current redistribution, homogenizing ion flux and blocking electron migration for stable high-voltage operation.
- The strategy is universally validated across sodium superionic conductor-type Li_1.3_Al_0.3_Ti_1.7_(PO_4_)_3_, garnet-type Li_7_La_3_Zr_2_O_12_, and perovskite-type Li_3_xLa_2_/_3-x_TiO_3_, providing a general strategy for engineering stable ceramic electrolytes.

**Supplementary Information:**

The online version contains supplementary material available at https://doi.org/10.1007/s40820-026-02347-w.

## Graphical abstract

**Figure:**
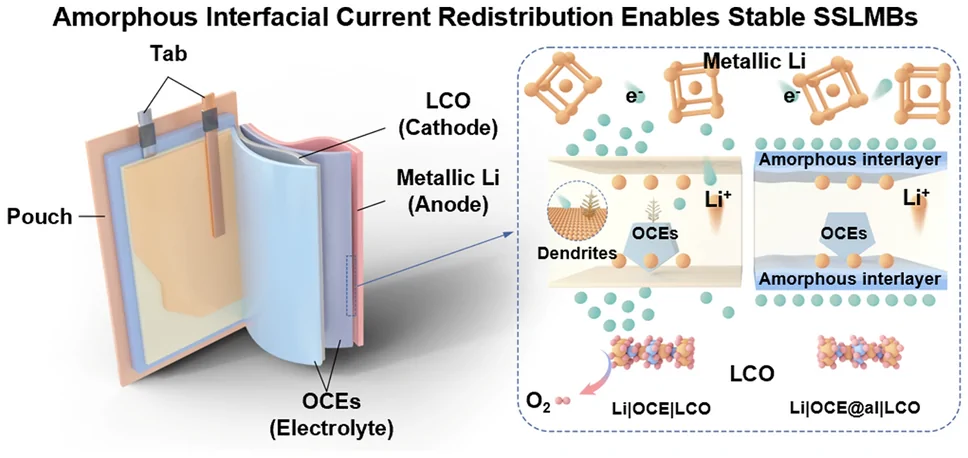

## Introduction

Oxide ceramic electrolytes (OCEs, e.g., sodium superionic conductor (NASICON), perovskite and garnet) offer inherent nonflammability, wide electrochemical windows, and high thermal stability, positioning them as promising candidates for solid-state lithium metal batteries (SSLMBs) [1–3]. However, interfacial instability in OCE-based SSLMBs is often attributed to chemical incompatibility between the electrolyte and lithium metal or mechanical failure induced by volume changes and dendrite penetration.

To mitigate interfacial failure, extensive efforts have focused on engineering the electrode–electrolyte interface by constructing protective interface layers, such as compositing OCEs with polymer [4–7] or decorating its surface with inorganic coatings [8–10]. The polymer composite strategy employs a “soft” organic phase to overcome the “rigid” nature of the ceramic, primarily by enhancing interfacial contact and mechanical adaptability [11]. In contrast, the inorganic coating approach acts as a sophisticated “buffer zone” and “interfacial modifier,” designed to suppress chemical side reactions and facilitate ion transport across the interface. Despite the enhanced stability from introducing new interfacial chemistries, these strategies simultaneously create more complex, multi-component interfaces. Within these engineered interphases, a convoluted interplay of chemical, electrochemical, and microstructural factors obscures the primary failure mechanism. The atomic-scale origins of this instability remain a critical blind spot that limits practical deployment.

Here, we shift the paradigm from extrinsic modification to intrinsic structural engineering of OCEs themselves. A defining feature of high-temperature sintered polycrystalline OCEs is their dense network of grain boundaries (GBs), where the periodic lattice is disrupted and ion/electron migration is governed [12–15]. Through integrating multiscale experimental characterization and theoretical simulation, this work reveals that surface GBs function as bipolar interfacial hotspots, actively driving dual-interface degradation via two interconnected mechanisms. At the lithium metal side, GBs exhibit locally enhanced electronic states and reduced charge-transfer barriers, promoting detrimental electron leakage and Li^+^ flux focusing. This lowers barriers for Li dendrite nucleation and enables electron-leakage-driven reduction of OCEs, leading to dendrite propagation along the GB network (Mechanism I). At the cathode side, GBs as high-energy defects trigger lithium segregation and electron redistribution, resulting in heterogeneous electronic fluxes and localized current concentration. Such current hotspots induce substantial localized overpotential regions, triggering irreversible phase transformation and lattice oxygen release (Mechanism II). Critically, both mechanisms originate from the same atomic-scale property: GBs act as electronically active, reaction-accelerating sites.

**Fig. 1:**
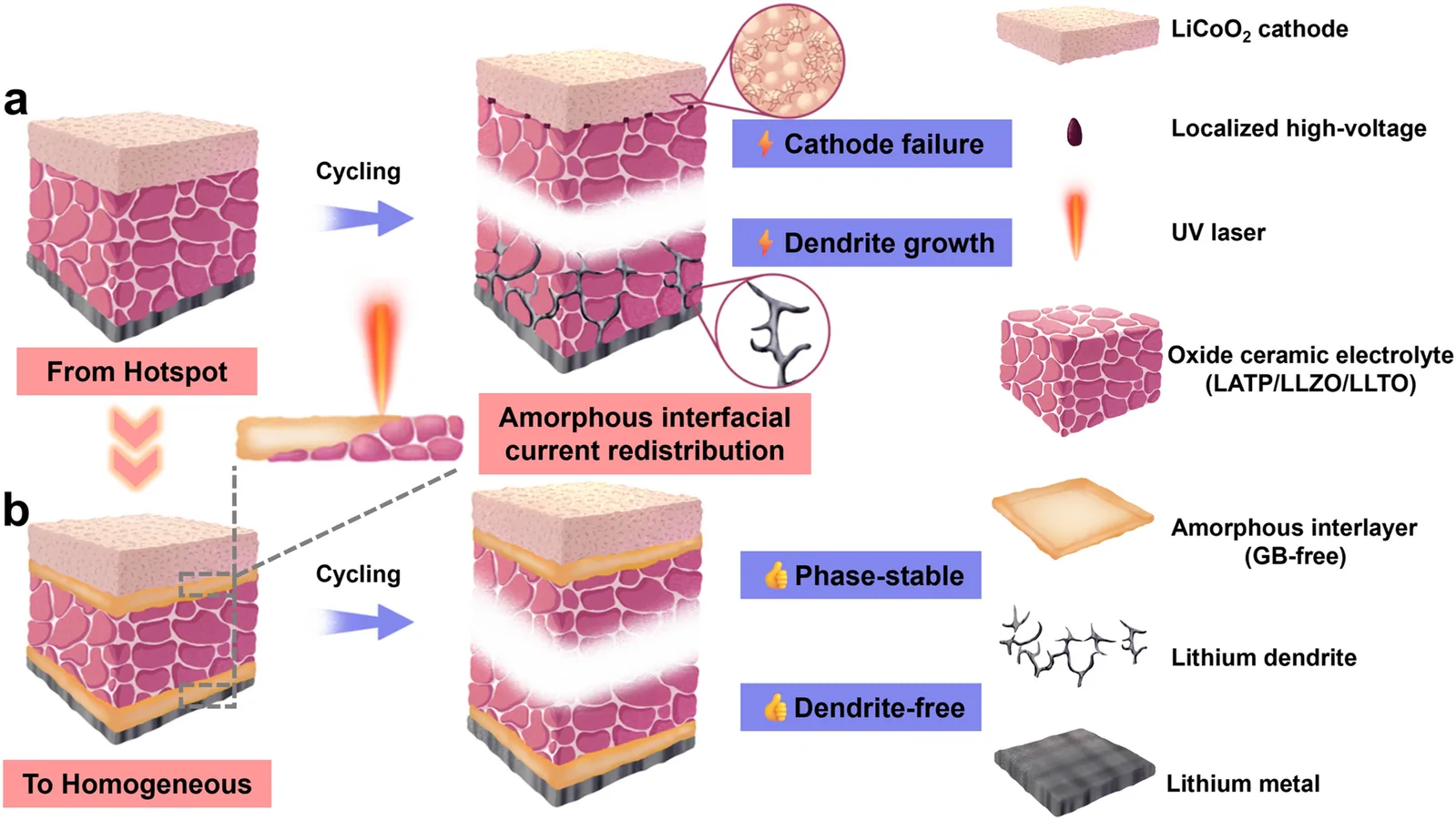
Schematic illustration of the amorphous interfacial current redistribution strategy, achieving the transition from hotspot to homogeneous in polycrystalline OCEs. **a** Hotspot stage: Pristine OCE exhibits localized overpotentials at the electrolyte-cathode interface induced by surface GBs, leading to structural degradation in part of the cathode. At the electrolyte-anode interface, dendrites infiltrate from the surface GBs. **b** Homogeneous stage: The laser-engineered amorphous interlayer eliminates surface GBs, achieving homogeneous current distribution and stable interfaces

To eliminate these active GB-derived hotspots, a rapid laser-driven amorphization technology with precise spatial control was employed to in situ construct a stable, thin and uniform amorphous interlayer (Fig. 1). First, surface GBs are identified as bipolar interfacial hotspots using a NASICON-type Li_1.3_Al_0.3_Ti_1.7_(PO_4_)_3_ (LATP) model system, which is chosen for its superior air stability and pronounced GB-mediated degradation [16–18]. Based on systematic analysis of the physicochemical properties of the amorphous interlayer and their correlation with electrochemical behavior, the electron-blocking and current-homogenizing functions of this disordered structure are validated. Building on this mechanistic understanding, effective dendrite suppression and cathode protection are demonstrated. Finally, these mechanistic insights are translated into substantially enhanced electrochemical performance in both Li symmetric cells and LiCoO_2_ (LCO) full cells, with the generality of this strategy further validated on garnet-type Li_7_La_3_Zr_2_O_12_ (LLZO) and perovskite-type Li_3x_La_2/3-x_TiO_3_ (LLTO). This stepwise progression from hotspot identification to performance demonstration and generality validation establishes “hotspot to homogeneous” as a unifying strategy for stabilizing polycrystalline OCEs.

## Experimental Section

### Materials

Commercial Li_1.3_Al_0.3_Ti_1.7_(PO_4_)_3_ (LATP), Li_7_La_3_Zr_2_O_12_ (LLZO), and Li_3x_La_2/3-x_TiO_3_ (LLTO) powders were purchased from Canrd Technology Co., Ltd. (China). LiCoO_2_ (LCO), Super P conductive carbon, poly(vinylidene fluoride) (PVDF), and N-methyl-2-pyrrolidone (NMP) were also obtained from the same supplier. A liquid electrolyte consisting of 1 M LiPF_6_ in ethylene carbonate/dimethyl carbonate (EC/DMC, 1:1 by volume) containing 5 vol% fluoroethylene carbonate (FEC) and a commercial electrolyte (LB-726) were supplied by Suzhou DodoChem Technology Co., Ltd. (China). All chemicals were used as received without further purification.

### Materials Synthesis

#### Preparation of Oxide Ceramic Electrolyte (OCE) Pellets

Fabrication of LATP, LLZO, and LLTO pellets: Commercial LATP, LLZO, and LLTO powders were each directly pressed into pellets using a 16 mm diameter mold under a pressure of 10 MPa for 1 min. The resulting green pellets were then sintered under different conditions: LATP pellets were sintered at 950 °C for 6 h, LLZO pellets at 1100 °C for 6 h, and LLTO pellets at 1150 °C for 6 h, all with a heating rate of 5 °C min^−1^ in air. After sintering, all pellets were naturally cooled to room temperature inside the furnace.

#### Preparation of Laser-Treated OCE Pellets

Laser-treated LATP pellets were fabricated from sintered LATP pellets using a high-precision ultraviolet (UV) laser (Diaotu UV Laser Marking System with a wavelength of 355 nm). The key parameters of the laser were set as follows: transition speed at 300 mm s^−1^, operating current at 1.0 A, and Q-pulse width at 1.0 μs. Based on the Gaussian distribution of laser energy, the line width of the laser beam was set as 5 μm to obtain a uniform energy distribution [19]. To investigate the properties of the samples obtained at different laser energies, the pulse repetition frequency of the laser varied to 50, 40, and 30 kHz to obtain the LATP pellets treated with different laser parameters. The numerical relationship between pulse repetition frequency and laser output power can be seen in Table S1. Following the laser process, the pellets were cleaned using a compressed nitrogen duster. LLZO and LLTO pellets were also subjected to surface processing under the same laser parameters at 40 kHz.

## Results and Discussion

### Laser-Induced Amorphization for GB-Free Surface

**Fig. 2:**
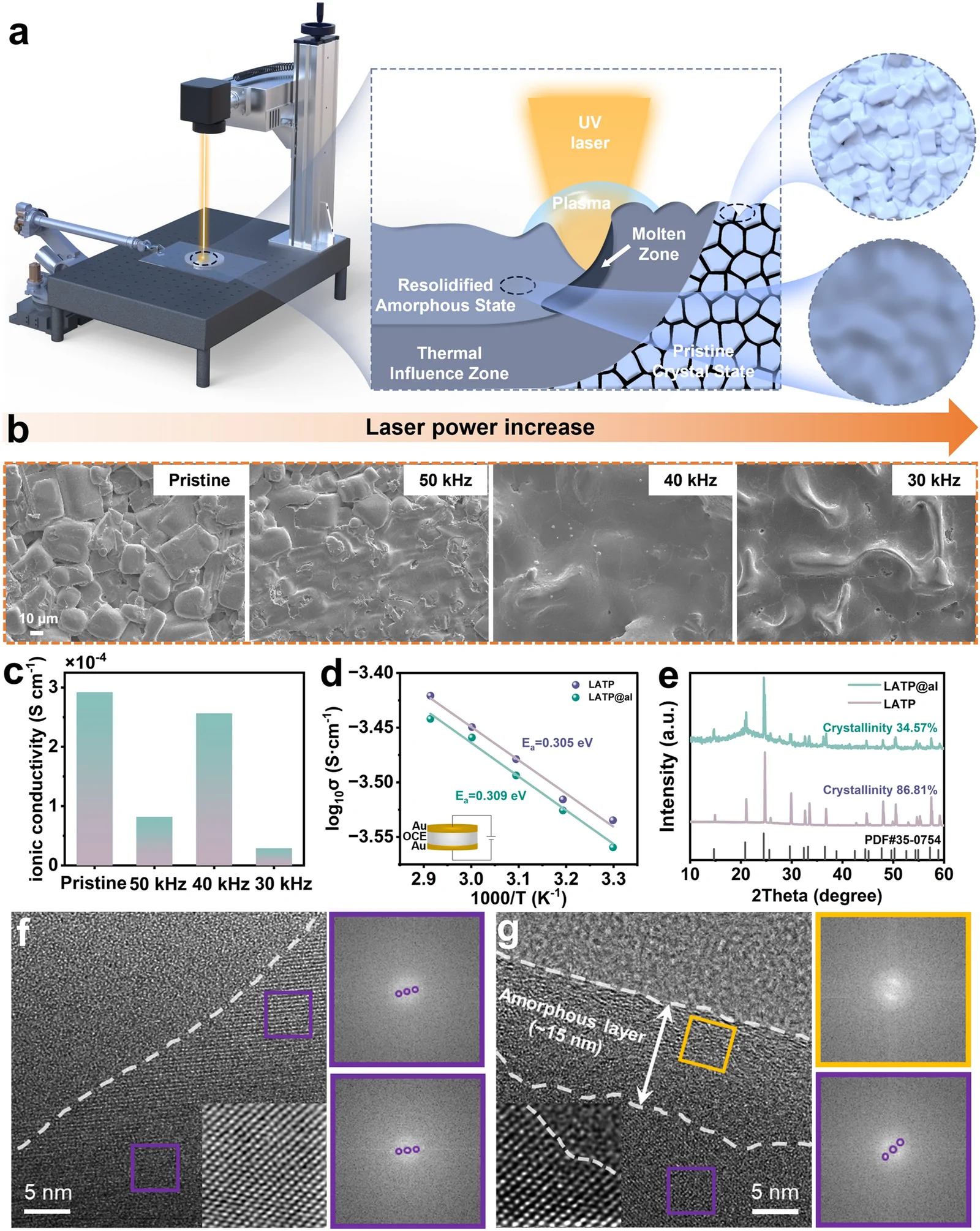
Schematic and characterization of the amorphous interlayer on LATP fabricated via laser ablation. **a** Illustration of the in situ laser-based amorphous interlayer fabrication process. **b** SEM image of the LATP surface after ablation with progressively increasing laser power and frequency (inset). **c** Ionic conductivity of pristine LATP and LATP treated under three laser parameter sets. **d** Arrhenius plots of LATP and LATP@al. **e** XRD patterns of before (LATP) and after (LATP@al) amorphous interlayer formation. High-resolution TEM images show microsrtucture of pristine LATP **f** and LATP@al **g** with corresponding HAADF-STEM image and FFT patterns

The amorphous interlayers on LATP pellets were in situ fabricated via a rapid UV laser treatment (Video S1 and Fig. S1). As illustrated in Fig. 2a, the LATP pellet experiences a rapid laser ablation, generating extreme localized temperatures confined to micrometer-scale regions via photothermal energy conversion (quantitatively analyzed in Fig. S2). This ultrafast thermal process triggered a cascade of non-equilibrium structural transformations: transient surface melting generated a highly disordered liquid phase, while laser-induced plasma formation drove multidirectional atomic redistribution. The subsequent rapid quenching kinetically bypassed recrystallization, freezing the melt into a glassy state before long-range order could be reestablished [20]. This kinetic arrest of the disordered configuration yielded a continuous, GB-free amorphous layer on LATP [21, 22]. The LATP pellets were treated with a gradient of laser pulse energy densities, precisely controlled from 10.06 to 19.31 J cm^−2^ by decreasing the pulse frequency from 50 to 30 kHz (see Table S2 for detailed parameters).

The morphology evolution of LATP pellets under varying laser energies was observed using scanning electron microscopy (SEM). Figure 2b shows that untreated pellets exhibited a rough surface formed by the accumulation of particles. In contrast, a smooth surface with completely melted GBs was obtained after laser treatment at 40 kHz. The pellet treated at 40 kHz (denoted as LATP@al) exhibits a smooth, densified surface, with a reduction in surface porosity from 14.5% (pristine LATP) to 3.2% (LATP@al) (Fig. S3). At lower energy inputs (50 kHz), only partial GBs melting occurred, leaving substantial fractions of the surface in its original polycrystalline state. However, excessively high energy inputs at 30 kHz caused overmelting, resulting in a rugged surface morphology.

As shown in Figs. 2c, d and S4, LATP@al exhibited the highest conductivity (2.56 × 10^–4^ S cm^−1^) among three laser‑treated samples, and this value is only slightly lower than that of pristine LATP (2.92 × 10^–4^ S cm^−1^), as measured through electrochemical impedance spectroscopy (EIS). The lower conductivity observed at 50 kHz can be attributed to the incomplete surface melting, which leaves the majority of grain boundaries intact and introduces contact voids at the electrode–electrolyte interface, thereby degrading the measured ionic transport. The lowest conductivity, observed at 30 kHz, results from the rugged, uneven surface caused by overmelting, which severely impairs solid–solid interfacial contact. Laser energy fluence therefore governs the quality of the amorphous layer and the resulting interfacial contact. Critically, all measured conductivities remain on the order of 10^–4^ S cm^−1^, indicating that the laser‑induced surface amorphization does not significantly alter the overall ionic transport. The excellent preservation of ion conduction is further evidenced by the nearly identical activation energies of LATP@al (0.309 eV) and pristine LATP (0.305 eV), confirming that the ultrathin amorphous layer presents no substantial barrier to Li^+^ transport.

Microstructural analysis reveals key differences between pristine and laser-modified LATP. X-ray diffraction (XRD) patterns demonstrate pristine LATP possesses perfect NASICON crystallinity (Fig. 2e). In contrast, the laser-treated LATP@al exhibits broadened diffraction peaks, indicating the loss of translational periodicity and the formation of a short-range-ordered surface layer consistent with amorphization. This structural disordering is further corroborated by Raman spectroscopy (Fig. S5), where LATP@al shows peak broadening and intensity attenuation relative to pristine LATP. These spectral features arise from the breakdown of selection rules in the disordered network, where the loss of long-range symmetry relaxes momentum conservation, enabling a broader continuum of vibrational states and thus a more uniform distribution of Raman activity across phonon modes [23]. Cross-sectional specimens of LATP and LATP@al were prepared by focused ion beam (FIB) milling for multimodal transmission electron microscopy (TEM) analysis. As illustrated in Fig. 2f, g, laser ablation successfully induced the formation of a thin (15 nm) and uniform amorphous surface interlayer on LATP@al. High-angle annular dark-field scanning TEM (HAADF-STEM) reveals the sharp amorphous-crystalline interface (inset), with fast Fourier transform (FFT) analysis confirming coexistence of disordered surface and bulk NASICON structure (R-3c space group), validating surface amorphization while preserving bulk crystallinity. From a manufacturing perspective, this ambient-air, high-speed laser process (~ 15 s cm^−2^) avoids vacuum deposition and multi-step chemical treatments, offering a scalable route for industrial integration.

### Electron-Blocking and Current-Homogenizing Functions

**Fig. 3:**
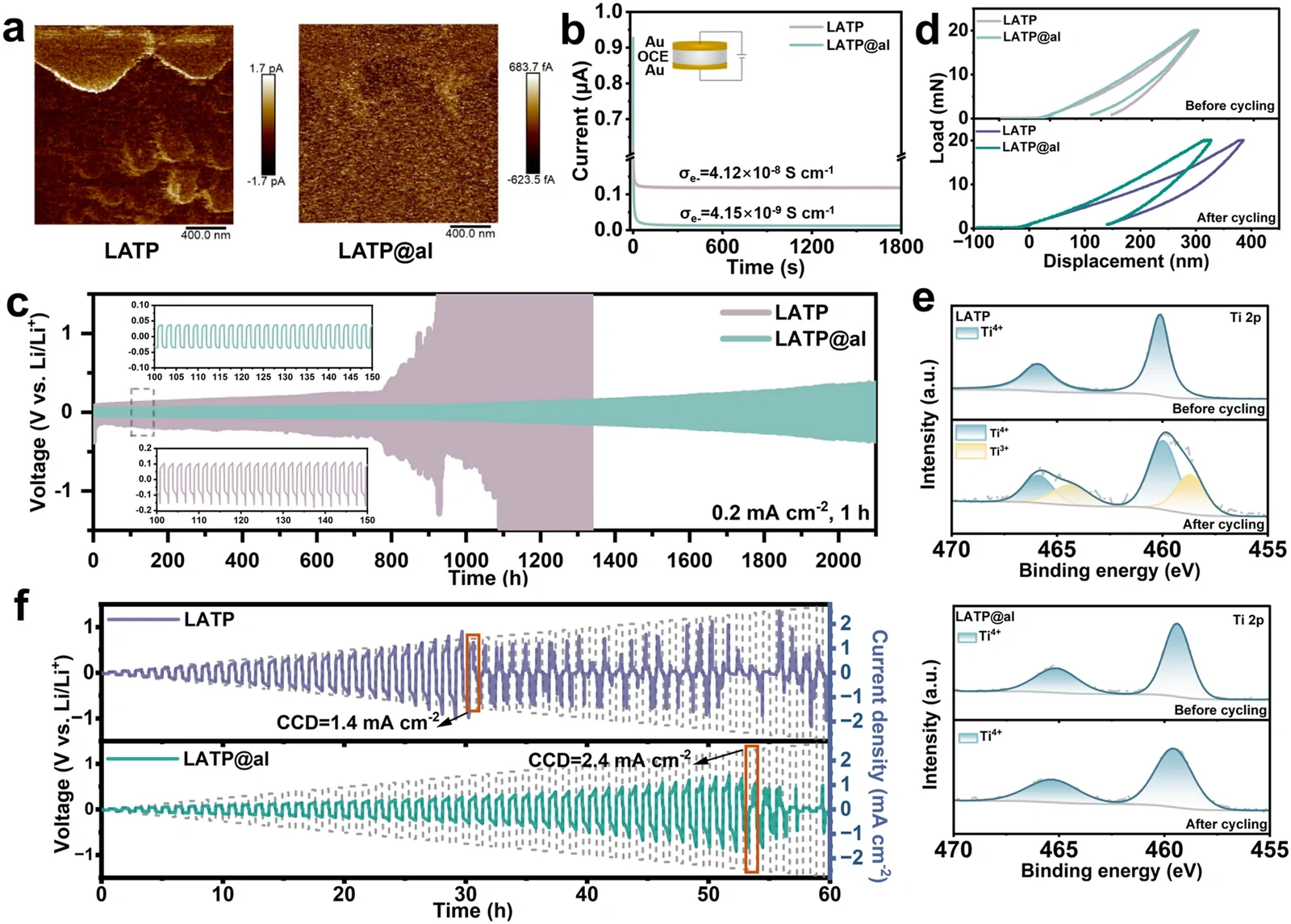
Electronic barrier properties and the interfacial stability of the amorphous interlayer. **a** The TUNA current maps of the LATP and LATP@al surfaces. **b** Electronic conductivity of LATP and LATP@al measured in Au | OCE | Au cell configuration, 150 mV polarization and r.t conditions. Performance of Li symmetric cell. **c** Durability comparison of LATP and LATP@al under Li symmetric cell configuration. **d** Surface nanoindentation load–displacement curves and **e** Ti 2*p* XPS spectra of LATP and LATP@al before and after 80 cycles. **f** Critical current density measurement of Li symmetric cell at R. T. (room temperature)

To elucidate the mechanistic correlation between surface structural modifications and charge transport properties, the electron flux characteristics of the amorphous layer on LATP@al were evaluated using tunneling atomic force microscopy (TUNA). Surface topography analysis (Fig. S6) reveals roughness decreased from 3.61 nm (LATP) to 0.577 nm (LATP@al) after laser treatment. Current mapping in Fig. 3a shows that the LATP surface exhibits localized current aggregation along GBs, while LATP@al exhibits a uniform current distribution across its surface. This implies that GBs serve as preferential electron conduction pathways, enabling localized charge accumulation. Quantitatively, the average current measured on LATP@al (0.104 × 10^–3^ pA) was three orders of magnitude lower than that on LATP (0.335 pA). This significant suppression of electronic conductivity originates from the amorphous layer’s disordered atomic framework, which introduces dense electron scattering centers that localize charge carriers and disrupt long-range electronic transport [24, 25]. This finding was corroborated by direct current (DC) polarization measurements (Fig. 3b), which confirms electronic conductivity reduction from 4.12 × 10^–8^ to 4.15 × 10^–9^ S cm^−1^. These results collectively confirm that the GB-free amorphous interlayer realizes the critical transition from hotspot-driven electronic leakage to homogeneous, electron-blocking transport, a key functionality of amorphous interfacial current redistribution.

The interfacial stability with the lithium metal anode was evaluated through galvanostatic cycling tests at 0.2 mA cm^−2^ in Li symmetric cells. As shown in Fig. 3c, after 100 h of cycling, the Li | LATP | Li exhibited a significantly higher overpotential (0.154 V) compared to the Li | LATP@al | Li (0.035 V). This difference indicates that the GB-free surface effectively homogenizes Li^+^ flux, eliminating localized current constriction and concentration polarization. Moreover, this performance divergence amplifies during extended cycling. The LATP system exhibited a continuously increasing overpotential, culminating in a surge beyond 1.0 V after 800 h. Satisfyingly, Li | LATP@al | Li cell maintained exceptional stability for over 2000 h without failure, confirming the GB-free surface effectively enhances interfacial stability with lithium metal. A comprehensive comparison of cycle stability and critical current density with representative literature (Table S3) further underscores the performance advantage of this amorphous interfacial current redistribution strategy.

The nano indentation technique was used to quantify the mechanical stabilization mechanism [26, 27]. In Fig. 3d, the LATP@al exhibits higher mechanical properties, with Young’s modulus (E) and hardness (H) values of ~ 92.35 and ~ 7.75 GPa, respectively, compared to those of crystalline LATP (~ 89.20 and ~ 8.17 GPa). Notably, the GB-free surface enables LATP@al to retain its superior mechanical properties after cycling, with E and H values of ~ 79.49 and ~ 6.41 GPa, respectively. In contrast, GB-rich surface exhibited significant mechanical degradation (reduced modulus ~ 61.38 GPa, hardness ~ 5.01 GPa), attributed to detrimental side reactions at the LATP | Li interface. In brief, surface GBs exacerbate the progressive contact loss between LATP and the lithium metal anode caused by reduction reaction by-products accelerates the increase in interface impedance. The amorphous interlayer with superior mechanical properties can effectively mitigate this issue, allowing Li | LATP@al | Li to demonstrate outstanding long-cycle stability. X-ray photoelectron spectroscopy (XPS) reveals critical differences in surface chemical stability. While both LATP and LATP@al show similar Ti^4+^ signatures initially (Fig. 3e), pristine LATP develops distinct Ti^3+^ peaks at 464.4 and 458.7 eV after 80 cycles, confirming continuous Ti^4+^ reduction by lithium metal. In contrast, LATP@al maintains unchanged Ti^4+^ states, verifying the GB effect on the pristine LATP surface introduce defect-derived electronic states and local potential inhomogeneity, thereby enhancing electron leakage and continuously driving parasitic interfacial reactions and degradation [28, 29]. Electrochemically, LATP@al sustains a higher critical current density of 2.4 mA cm^−2^ compared to 1.4 mA cm^−2^ for LATP, demonstrating superior dendrite suppression (Fig. 3f). Furthermore, the stable overpotential increment of LATP@al based Li symmetric cell across varying current densities further evidence enhanced interfacial stability (Fig. S7).

### Current Redistribution and Li Deposition Dynamics

**Fig. 4:**
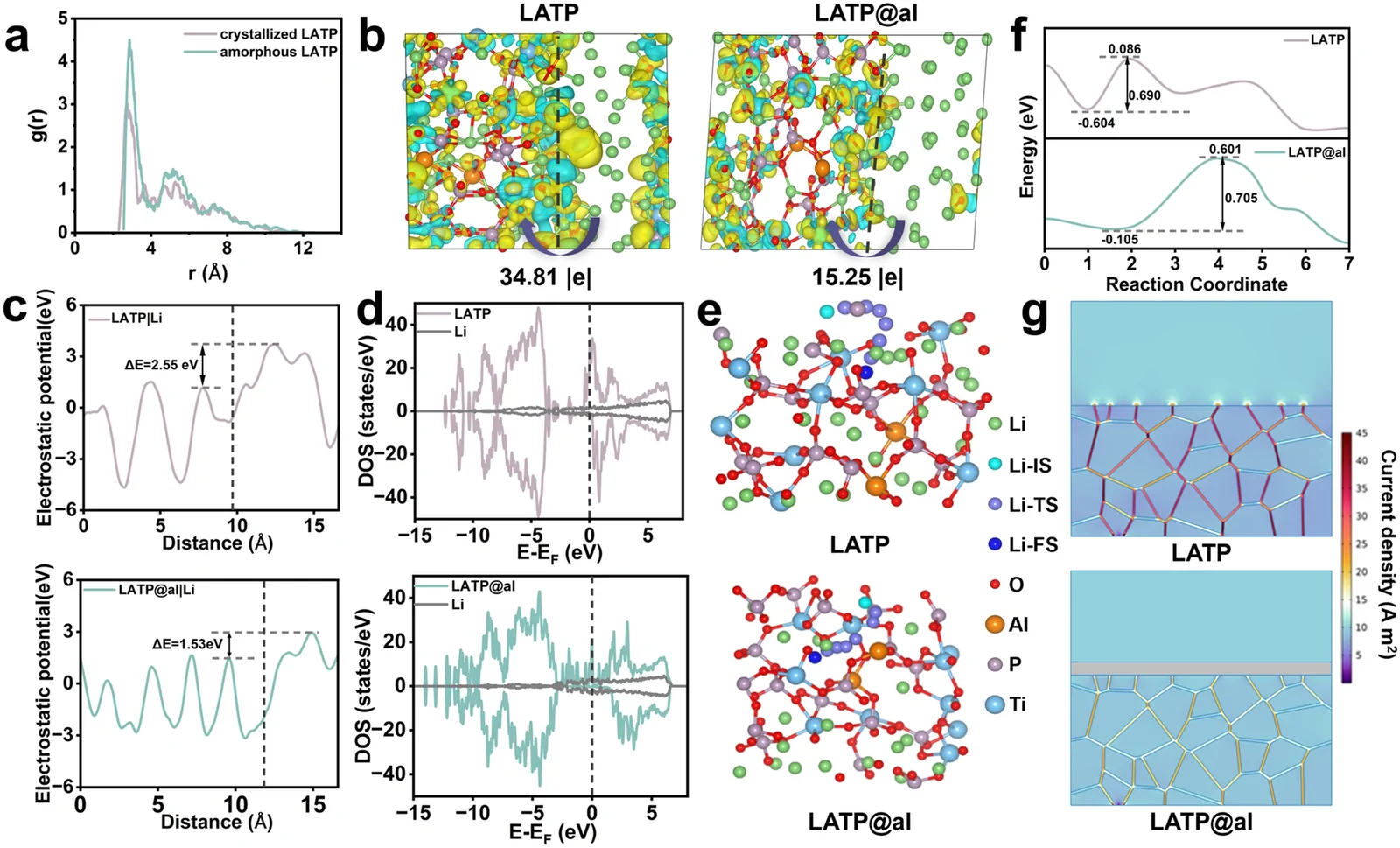
Simulation for electronic and ionic migration properties. **a** Comparison of the Li–Li radial distribution functions of crystalline and amorphous LATP. **b** Charge transfer of Li | LATP interface and Li | LATP@al. Blue color represents charge dissipation and yellow color indicates charge aggregation, the isosurface value is 0.005 e Å^−3^. **c** 1D electrostatic potential distribution at the corresponding interfaces along the c-axis direction of the lattice. **d** DOS of the corresponding interfaces. **e** Li migration path in LATP and LATP@al, with the transition state (Li-TS, lavender) highlighted. Other elements are labeled on the right. This path corresponds to the highest energy barrier during Li migration in **f**. **g** Finite element simulation results of current distribution at the LATP | Li and LATP@al | Li interfaces

The origin of interfacial stability was first probed by reproducing the laser-induced amorphization process using ab initio molecular dynamics (AIMD) simulations, which captured the transient thermal gradient characteristic of the laser (Video S2). The radial distribution function (RDF) analysis in Fig. 4a reveals distinct structural differences between pristine LATP and modified LATP@al systems. The Li–Li peak broadening in LATP@al indicates a wider distribution of interatomic distances, resulting from disrupted lattice periodicity and enhanced structural disorder. This confirms successful amorphous phase generation [30], establishing LATP@al and LATP (Fig. S8) as validated models for subsequent first-principles density functional theory (DFT) simulations.

The electronic barrier effect of the laser-induced amorphous interlayer was verified by DFT calculations. Charge distribution analysis (Fig. 4b reveals significantly reduced charge transfer at the LATP@al | Li interface (15.25 e^−^) versus LATP | Li (34.81 e^−^). This reduction stems from disordered atomic arrangements weakening interfacial electronic interactions, demonstrating effective electron-blocking capability. The interfacial electrostatic potential barrier decreases from 2.55 (LATP | Li) to 1.53 eV (LATP@al | Li) along the c-axis, demonstrating effective suppression of electron injection from Li to OCE (Fig. 4c). Spin-resolved density of states (DOS) analysis (Figs. 4d and S9) reveals fundamental electronic structure modifications at the interface. LATP | Li exhibits metallic-like spin-up partial DOS (PDOS) near the Fermi level (E_F_) and a 1.58 eV spin-down gap, while LATP@al | Li shows disrupted spin-up PDOS continuity with diminished peaks at E_F_ and a substantially wider spin-down band gap of 4.26 eV, reducing electron concentration. The calculated Li^+^ migration barrier increases marginally from 0.690 eV (LATP) to 0.705 eV (LATP@al) (Fig. 4e, f), indicating that the disordered surface structure preserves efficient Li^+^ transport pathways. This selective functionality, characterized by strong electron blocking with negligible ionic impedance, originates from the fundamentally different transport mechanisms in disordered media: electrons are localized by scattering centers, whereas ions migrate through accessible coordination sites despite the loss of long-range order. Therefore,laser-induced surface amorphization eliminates GB-mediated electron leakage without compromising the ionic conductivity of the crystalline framework, forming an ideal interfacial electronic barrier for stable electrode–electrolyte interface.

The finite element analysis elucidates the contrasting current distribution modes at the two interfaces. As shown in Fig. 4g, the pristine LATP exhibits uneven current distribution with localized hotspots at the GBs (validated by TUNA), confirming preferential electron conduction along defects. Conversely, LATP@al demonstrates uniform current distribution, quantitatively attesting to the effective suppression of GB-mediated electronic conductivity. These modeling findings elucidate the fundamental mechanism through which the amorphous surface interlayer functions as an electron-regulating “sluice gate”, obstructing preferential dendrite infiltration pathways by disrupting the GB network. This effect of electron redistribution homogenizes the downstream current distribution, subsequently suppressing localized lithium deposition.

In situ optical observations (Figs. S10 and S11) reveal that under high current density (~ 6 mA cm^−2^), the uneven current distribution on LATP triggers continuous localized lithium deposition. The resulting concentrated stresses induce significant crack propagation. Additionally, electron leakage promotes Li^+^ reduction within the OCE bulk, generating lithium dendrites. These dendrites progressively penetrate the electrolyte, mechanically compromising its structural integrity and ultimately causing failure. In contrast, the amorphous interlayer in LATP@al systems effectively suppresses localized deposition and dendritic nucleation through dual-function barrier mechanisms, thereby maintaining electrolyte stability.

### Mitigating GB-Induced Dendrite Penetration

**Fig. 5:**
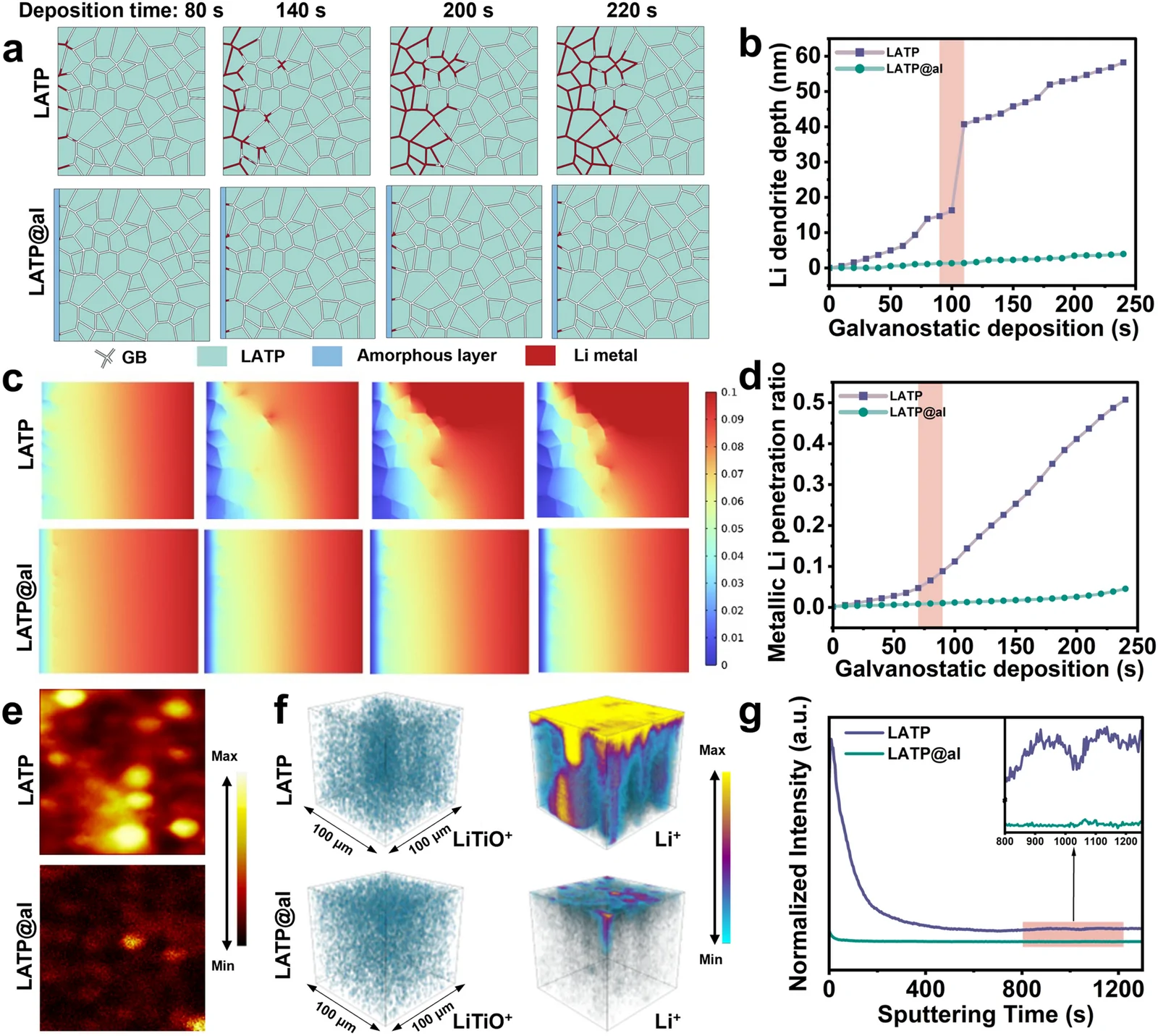
Evidence of spatial evolution of lithium dendrites in OCEs. **a** Phase-field modeling of Li dendrite growth and **b** electric potential distributions during 240 s galvanostatic deposition within a 100 × 100 μm^2^ of LATP and LATP@al. The dynamic evolution of **c** lithium dendrite penetration depth and **d** total metal lithium content during galvanostatic deposition within OCEs. Li^+^ distribution within OCEs after 200 h cycled in Li | OCEs | Li cell: **e** ToF–SIMS Li^+^ distribution maps (100 × 100 μm^2^ analysis area, 1300 s Ar^+^ sputtering) of LATP and LATP@al. **f** 3D reconstructions of LiTiO^+^ and Li^+^ spatial distributions. **g** The corresponding depth profiles of Li^+^ intensity versus sputtering time

The mechanism of anode degradation driven by dendrite penetration initiating at surface GB is elucidated through simulations of its spatiotemporal evolution and the associated interfacial polarization dynamics during deposition (Fig. 5a, Video S3). In pristine LATP, at 140 s, significant localized lithium dendrites formation inside LATP can be observed, which is attributed to electron-mediated Li^+^ reduction along preferential GB pathways, leading to independent nucleation. This corresponds to a sudden surge in the dendrite depth over time observed in the LATP curve in Fig. 5b. In contrast, the amorphous interlayer on LATP@al blocks electrons, kinetically limiting Li^+^ reduction and eliminating internal nucleation sites for dendrites. The overpotential distribution presented in Fig. 5c indicates the occurrence of sudden voltage drops within the LATP system, which are primarily attributed to electron leakage-induced Li^+^ reduction at GBs. The infiltration process in LATP@al exhibits a slower progression. This aligns with the observed evolution of dendrite infiltration shown in Fig. 5d.

Leveraging the sensitivity of time-of-flight secondary ion mass spectrometry (ToF–SIMS) to lithium metal clusters, metallic lithium (Li^0^) ionizes to form Li^+^ under ion bombardment. Thereby, the intensity of the Li^+^ signal is directly correlated with the density of Li^0^ in localized areas [31]. Chemical mapping of Li^+^ (Fig. 5e) reveals heterogeneous Li^+^ distribution within the near-surface region of LATP, with localized hotspots corresponding to the defects/GBs nucleation sites, whereas LATP@al exhibits homogeneous Li^+^ distribution. Comparative 3D reconstructions (Fig. 5f) confirm uniform LiTiO^+^ spatial profiles across both systems, while revealing localized Li^+^ accumulation exclusively in LATP. Furthermore, the variation in Li^+^ distribution at different depths in Video S4 reveals that Li^+^ clusters in LATP bulk arise not only from surface propagation, but also through independent Li^0^ nucleation at internal defect sites. By contrast, Li^+^ in LATP@al aggregates only on the surface, reaffirming the role of the amorphous electronic barrier interlayer in effectively inhibiting internal dendrite nucleation. Depth profiling (Fig. 5g) highlights distinct Li distribution behaviors: LATP shows strong surface Li accumulation, evidenced by peak Li^+^ intensity during initial sputtering and corroborated by SEM observation of surface lithium clusters on cycled LATP pellet (Fig. S12). Beyond the surface region, the bulk Li^+^ concentration of LATP remains higher intensity than LATP@al after stabilization (> 400 s sputtering), with persistent minor fluctuations that may be indicative of localized dendritic formations. These fluctuations are in accordance with phase-field simulations predicting dendrite nucleation at GBs. Figures S13 and S14 present cross-sectional Auger electron spectroscopy (AES) elemental maps of cycled LATP and LATP@al electrolytes extracted from Li | OCEs | Li symmetric cells after 200 h of operation, respectively. These results reveals localized Li aggregates in LATP but a homogeneous, low-intensity Li distribution in LATP@al, confirming effective dendrite suppression.

The observations above reveal that dendrite penetration in polycrystalline LATP follows a sequential process initiated at surface GBs. First, surface GBs act as electronic hotspots, injecting electrons into the near-surface region and forming initial Li nuclei. Once these electrons are present in bulk, they further reduce migrating Li^+^, generating distributed internal Li deposits [32]. Subsequently, confined Li deposition generates high pressure, inducing tensile stress that drives crack growth and allows dendrites to penetrate the electrolyte [33]. In this framework, surface GBs serve as the critical entry point for electron leakage. By eliminating these GBs via laser-induced amorphization, the amorphous interlayer blocks electron injection at the initial step, thereby suppressing subsequent bulk nucleation and mechanical failure without altering bulk ionic conductivity.

### Suppressing Localized High-Potential Degradation at Cathode

**Fig. 6:**
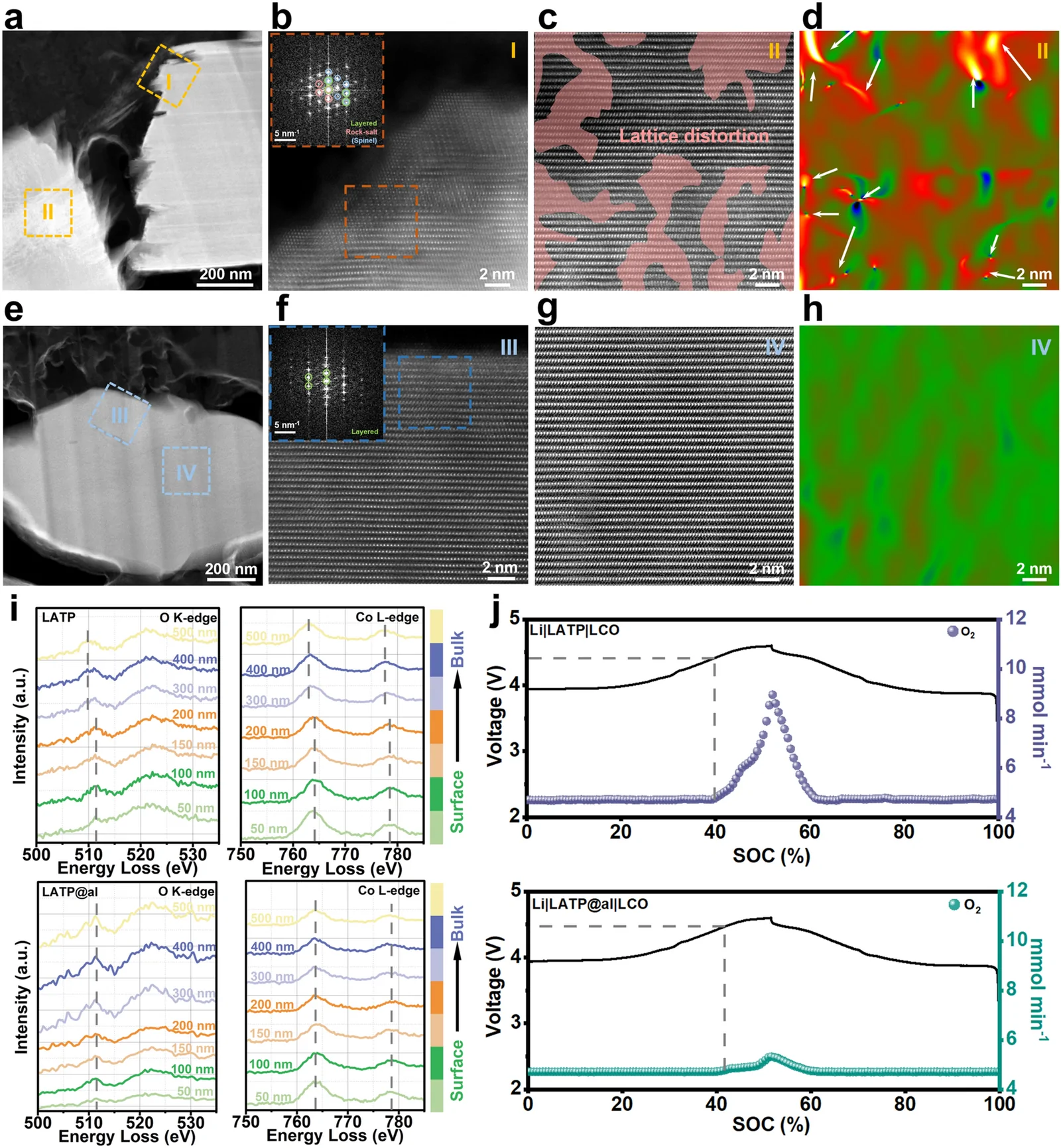
Comparative analysis of cathode | electrolyte interface stability. **a**-**j** Microstructure and chemical evolution after 100 cycles at 1 C: Low-magnification STEM images of the cross sections of LCO from cells with LATP (**a**) and LATP@al (**e**) electrolytes. **b**, **c** High-magnification HAADF-STEM images corresponding to the areas marked I and II in **a**, respectively. **f**, **g** Analogous high-magnification images of areas III and IV in **e**. FFT patterns insets in **b**, **f**. **d**, **h** GPA strain maps derived from images **c** and **g**, respectively. **i** EELS line scans from the surface to the center of the particles in **a** and **e**, revealing chemical profiles. **j** In situ DEMS analysis of Li | LATP | LCO and Li | LATP@al | LCO cells during the first charge to 4.6 V (vs. Li^+^/Li) at 0.1 C

The efficacy of the amorphous disordered interlayer in mitigating cathode degradation by removing surface GBs was evaluated in full-cell configurations paired with commercial LCO cathode. Compared to the severe cracking in LCO cycled with bare LATP (Fig. 6a), LCO particles protected by the LATP@al interlayer (Fig. 6e) maintain excellent structural integrity. Atomic-resolution HAADF-STEM imaging of a cracked region (area I, Fig. 6b) in the LATP-based LCO shows the coexistence of layered, rock-salt, and spinel phases, confirmed by the corresponding FFT pattern (inset), indicating irreversible phase transformation and structure degradation. In contrast, the LCO particle cycled with LATP@al (area III, Fig. 6f) retains a well-defined layered structure at the surface region. Further analysis of the bulk microstructure via geometric phase analysis (GPA) indicates significant local strain accumulation within the LATP-based LCO (Fig. 6c, d), likely driven by lattice distortion and phase inhomogeneity, which contributes to crack initiation. Conversely, the LATP@al-based sample exhibits minimal strain and uniform lattice (Fig. 6g, h), suggesting enhanced mechanical stability imparted by the amorphous interlayer. These structural observations collectively support the conclusion that surface GBs act as hotspots for detrimental phase transitions under localized high voltage.

Complementary EELS line-scan profiles from surface to bulk (Fig. 6i, acquisition area shown in Fig. S15) provide chemical insights [34]: at the O K-edge, a slight shift of the pre-peak toward higher energy loss near the surface of the LATP-based LCO suggests stronger oxygen-cation bonding, consistent with oxygen depletion and phase transformation. At the Co L-edge, a similar energy loss shift implies the presence of mixed Co^3+^/Co^4+^ valence states, likely associated with surface oxidation and local coordination changes. These chemical shifts are less pronounced in the LATP@al-based sample, indicating that the amorphous interlayer helps preserve the bulk electronic environment and mitigates surface degradation [35]. Furthermore, in situ differential electrochemical mass spectrometry (DEMS) analysis (Fig. 6j) of the Li | LATP | LCO configuration reveals significant O_2_ gassing, originating from current density inhomogeneity across the LATP surface. Localized hotspots develop interfacial potentials substantially exceeding the average cell voltage, thereby inducing oxygen evolution below the intrinsic stability limit of LCO. In contrast, the introduction of an amorphous interlayer in the Li | LATP@al | LCO system effectively homogenizes current distribution on LATP@al, eliminating localized voltage spikes and significantly suppressing oxygen release.

In situ galvanostatic EIS (GEIS) coupled with distribution of relaxation times (DRT) analysis was employed to probe interfacial impedance evolution of the Li | OCEs | LCO cell during the initial two cycles. The corresponding charge/discharge profiles of LATP and LATP@al are shown in Figs. S16 and S17, respectively. Figures S18 and S19 present a representative DRT deconvolution, where each relaxation time constant (τ) corresponds to specific electrochemical processes: τ_1_ (10^–7^-10^–4^ s) corresponds to the bulk resistance of the electrolyte (R_bulk_). τ_2_ (peak at 10^–4^-10^–3^ s) is attributed to the evolution of the solid electrolyte interphase (SEI) evolution governed by state-of-charge (SOC)-dependent lithiation/delithiation processes. τ_3_ (peak at 10^–3^-10^–2^ s) and τ_4_ (peak at 10^–2^-10 s) represent the charge transfer resistance at the Li | OCE interface (R_ct-Li_) and the LCO | interface (R_ct-LCO_), respectively [36]. The DRT profiles (Figs. S20-S22) exhibit pronounced SOC-dependent impedance characteristics, particularly in the low-frequency domain. The peak intensities quantified resistance contributions, while time constants reflected reaction kinetics. Notably, R_ct-LCO_ in Li | LATP | LCO significantly exceeded that in Li | LATP@al | LCO with a resistance over an order of magnitude higher, indicating severe interfacial degradation after high-voltage cycling. In contrast, LATP@al exhibited reduced R_ct-LCO_ and a shift toward lower τ-values, suggesting faster charge transfer kinetics. This improvement stems from the current-homogenizing effect of the amorphous interlayer, which mitigates localized high-voltage hotspots, suppresses interfacial side reactions, and enhances Li^+^ migration. Moreover, Ti^4+^ reduction by Li metal on LATP generates poorly conductive phases, degrading interfacial ion transport. The amorphous interlayer in LATP@al effectively inhibits this parasitic reaction, yielding lower R_ct-Li_. The stable interfacial impedance of LATP@al at identical SOC confirms enhanced electrode | OCE compatibility and charge transfer kinetics.

### Dual-Interface Stabilization for High-Voltage SSLMBs

**Fig. 7:**
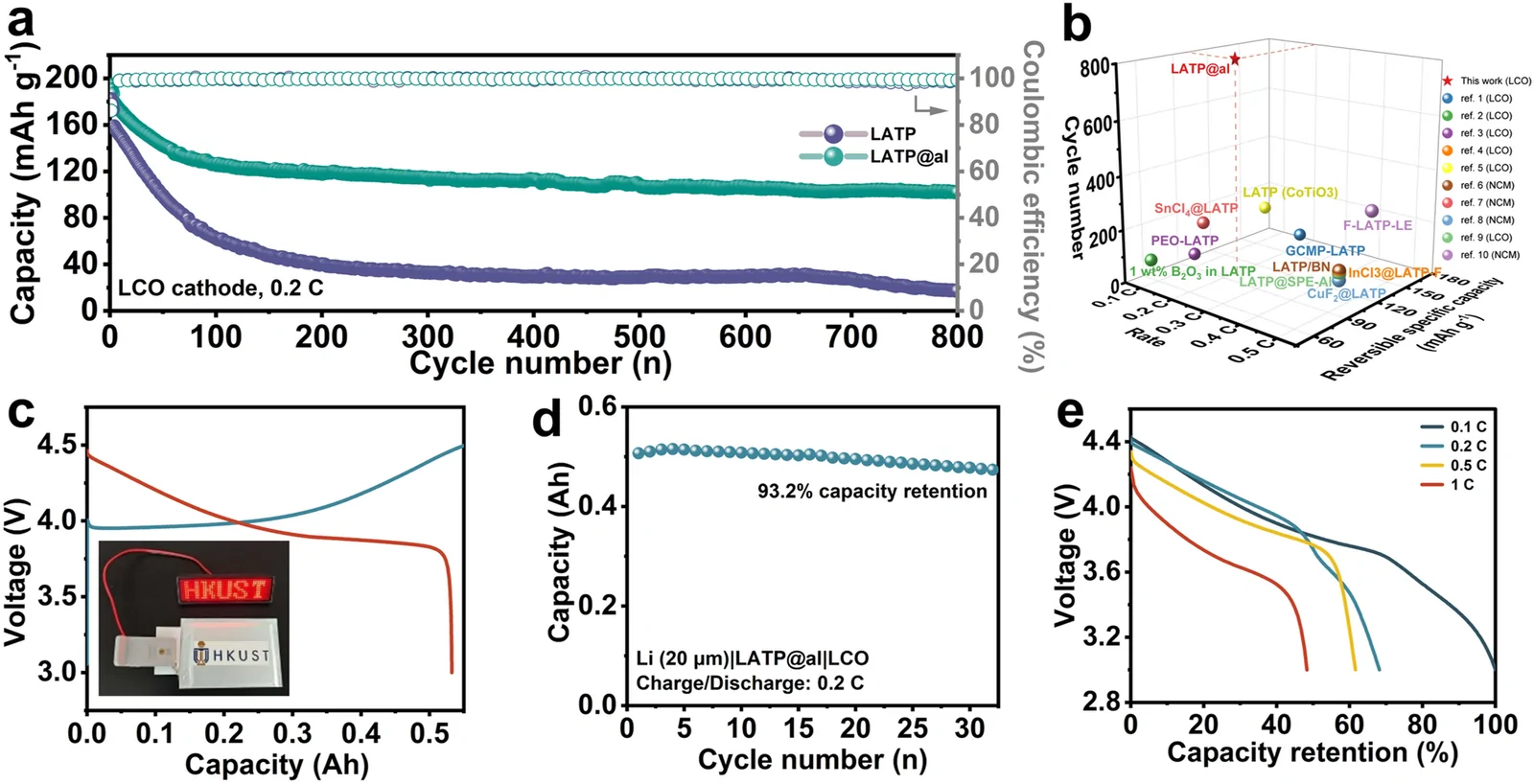
Electrochemical performance of SSLMBs paried with high-voltage LCO cathode. **a** Cycling performance of Li | LATP | LCO and Li | LATP@al | LCO cells at 0.2 C. **b** Performance comparison of this work with reported optimization strategies for LATP-based SSLMBs. **c** Initial charge–discharge profiles, **d** cycling stability and **e** rate capability of LATP@al-based pouch cells

This interfacial stabilization underpins the superior electrochemical performance in SSLMBs. Even under high-rate cycling at 2 C (1 C = 178 mAh g^−1^), the Li | LATP@al | LCO cell delivers a reversible capacity of 110.2 mAh g^−1^ (Fig. S23), significantly surpassing the mere 22.5 mAh g^−1^ achieved by the LATP-based counterpart. Furthermore, the Li | LATP@al | LCO cell demonstrates excellent cycling stability, delivering a reversible capacity of 101.9 mAh g^−1^ after 800 cycles (Fig. 7a), far exceeding the performance of its unmodified counterpart (17.2 mAh g^−1^). Based on the performance comparison (Fig. 7b, see Table S4 for detailed list), this work achieves an advanced level of stability in high-voltage SSLMBs, substantially outperforming previously reported modification strategies for LATP-based electrolytes. The superior performance is attributed to the amorphous interlayer that redistributes interfacial current, thereby eliminating GB-derived bipolar hotspots. This unique mechanism simultaneously homogenizes Li^+^ flux, blocks electron leakage, and suppresses both dendrite penetration and cathode degradation, thereby enabling stable operation under high-voltage conditions.

To further validate the practicality of laser-induced free-GB surface of LATP, pouch cells with a Li | LATP@al | LCO configuration were assembled. The pouch cell delivered an initial capacity of 532.6 mAh (Fig. 7c), stably cycled over 30 cycles with 93.2% retention finally (Fig. 7d, and maintained > 50% capacity retention across 0.1–1 C rates (Fig. 7e). During nail penetration tests, the solid-state Li | LATP@al | LCO pouch cell exhibited no thermal runaway, in stark contrast to the liquid electrolyte cell which showed a rapid temperature spike (> 2000 °C s^−1^) and violent combustion (Fig. S24 and Video S5). These results demonstrate that the amorphous interlayer enables intrinsically safe, thermal-runaway-free SSLMBs.

### Cross-System Generality Validation

For assessing the general applicability of this amorphous interfacial current redistribution strategy, laser treatment was extended beyond LATP to two representative OCE families: garnet-type LLZO and perovskite-type LLTO. To further confirm the surface structural evolution, XRD and SEM characterizations were performed on LLZO and LLTO before and after laser treatment (Figs. S25 and S26). For both electrolytes, laser treatment leads to distinct XRD peak broadening, indicating decreased surface crystallinity, while the corresponding SEM images reveal a transition from rough particle-aggregated surfaces to smooth, melt-resolidified morphologies. These features are consistent with those observed for LATP@al. The resulting LLZO@al and LLTO@al symmetric cells exhibited enhanced cycling stability compared to their pristine counterparts (Figs. S27 and S28), confirming that eliminating surface GB hotspots consistently improves interfacial stability across diverse polycrystalline OCEs. Moreover, laser-treated LLZO@al | LCO and LLTO@al | LCO full cells also exhibited variably but distinctly improved cycling stability (Figs. S29 and S30).

## Conclusions

In summary, this work identifies GB-induced electron leakage and localized current concentration as the atomic-scale origins of dual-interface failure in OCEs. To eliminate these active GB-derived hotspots, a laser-driven amorphization strategy was developed. The resulting amorphous interlayer homogenizes ion flux and blocks electron migration, enabling a critical transition from heterogeneous to homogeneous transport. For LATP, this amorphous interfacial current redistribution enables a critical current density of 2.4 mA cm^−2^, stable symmetric cell cycling for over 2000 h, and a reversible capacity of 101.9 mAh g^−1^ after 800 cycles in LCO full cells at 4.5 V, with pouch cells demonstrating intrinsic safety. This approach effectively decouples the otherwise intertwined degradation pathways. Beyond LATP, the effectiveness of this amorphous interfacial current redistribution strategy is further validated across garnet-type LLZO and perovskite-type LLTO, confirming its universality in stabilizing diverse polycrystalline OCEs. This work shifts the paradigm from managing degradation consequences to eliminating their structural origin, which provides a new route to durable, high-energy SSLMBs.

## Supplementary Information

Below is the link to the electronic supplementary material.Supplementary file1 (MP4 1896 kb)Supplementary file2 (MP4 1372 kb)Supplementary file3 (MP4 904 kb)Supplementary file4 (MP4 1108 kb)Supplementary file5 (MP4 2307 kb)Supplementary file6 (DOCX 52151 kb)
