# Supplementary material for: Hotspot to Homogeneous: Amorphous Interfacial Current Redistribution Enables Stable Solid-State Lithium-Metal Batteries
Source: Nanomicro Lett. 2026 Aug 27;19:40. doi: 10.1007/s40820-026-02347-w (PMC13522334; doi:10.1007/s40820-026-02347-w)
Supplement: Supplementary file 1 [file 40820_2026_2347_MOESM6_ESM.docx]

Supporting Information for

**Hotspot to Homogeneous: Amorphous Interfacial Current Redistribution Enables Stable Solid-State Lithium-Metal Batteries**

Cuiyun Yang^1^, Xupeng Lu^1^, Yexin Pan^1^, Qimeng Zhang^2^, Ruohan Yu^3^, Rongliang Yang^4^, Huan Liu^1^, Molong Duan^5^, Mitch Guijun Li^1, 6, 7,^ *, Ziyi Zhu^8,^ *, and Chenghao Yang^2,^ *

^1^ Division of Integrative Systems and Design, The Hong Kong University of Science and Technology, 999077, Hong Kong SAR, P. R. China

^2^ School of Environment and Energy, South China University of Technology, Guangzhou, 510006, P. R. China

^3^ The Sanya Science and Education Innovation Park of Wuhan University of Technology, Sanya, 572000, P. R. China

^4^ Department of Mechanical Engineering, City University of Hong Kong, Hong Kong SAR, P. R. China

^5^ Department of Mechanical and Aerospace Engineering, The Hong Kong University of Science and Technology, 999077, Hong Kong SAR, P. R. China

^6^ Department of Electronic and Computer Engineering, The Hong Kong University of Science and Technology, 999077, Hong Kong SAR, P. R. China

^7^ Center for Smart Manufacturing, The Hong Kong University of Science and Technology, 999077, Hong Kong SAR, P. R. China

^8^ School of Metallurgical and Energy Engineering, Kunming University of Science and Technology, Kunming, 650093, P. R. China

*Corresponding authors. E-mail: [mitchli@ust.hk](mailto:mitchli@ust.hk) (Mitch Guijun Li); [zyzhu23@kust.edu.cn](mailto:zyzhu23@kust.edu.cn) (Ziyi Zhu); [esyangc@scut.edu.cn](mailto:esyangc@scut.edu.cn) (Chenghao Yang)

# **S1 Experimental Section**

**S1.1 Testing battery assembly**

Cathode Fabrication: Cathode slurries were prepared using commercial LCO active materials, Super P carbon, and PVDF binder (8:1:1 mass ratio). Dry components were dispersed in NMP solvent via mechanical mixing, coated onto aluminum foil using doctor-blading, vacuum-dried for 12 h. The active material loading was controlled at $\sim$3 mg cm^-2^, and the dried electrodes were punched into 10 mm diameter disks.

Cell Configurations:

- Coin cells: 2032-type cells with LCO cathodes and LATP (LLZO/LLTO) pellets assembled in Ar-filled glovebox (H_2_O < 0.1 ppm, O_2_ < 0.1 ppm), 10 wt% commercial electrolyte (DoDoChem, LB-726) was added to wet the cathode interface.
- Symmetric cells: CR2032 with Li | OCE | Li configuration using 10 mm Li foils. To improve interfacial contact, 5 $\mu$L of 1 M LiPF_6_ in DMC:EC (1:1 vol%) with 5% FEC was used to wet the interface.
- Blocking cells: Au | OCE | Au with 500 nm sputtered gold electrodes.
- Soft-pouch cells: Cells (nominal 0.5 Ah capacity) were assembled by stacking two cathodes (LCO, areal loading: 38 mg cm^-2^) and three anodes (20 $\mu m$ thick lithium foil) within aluminum-laminated pouches. 5 wt% commercial electrolyte (DoDoChem, LB-726) was injected prior to sealing.

**S1.2 Electrochemical testing protocol**

The ionic conductivity of different OCEs was measured using EIS in Au | OCE | Au blocking cells. The EIS measurement was performed with a PARSTAT electrochemical workstation over a frequency range of 0.1 Hz to 1 MHz and an alternating current (AC) amplitude of 10 mV.

The electronic conductivity ($\sigma$_e_) was determined using Au | OCE | Au blocking cells. The value was calculated using the following equation:

|  | $\sigma_{e}=\frac{1}{\rho}=\frac{I_{ss}l}{US}$ | S1 |
| --- | --- | --- |

where *I*_ss_ denotes the steady-state current, *l* represents the pellet thickness of the OCE, *U* indicates the applied voltage (150 mV), and *S* corresponds to the effective contact surface area.

The critical current density (CCD) was determined using an initial current density of 0.05 mA cm^–2^, incrementing by 0.05 mA cm^–2^ per step at room temperature. Each charge/discharge step lasted 30 minutes. Galvanostatic cycling (including cycle performance and rate capability testing) and the CCD measurements were performed on a LAND battery test system.

*In situ* galvanostatic current electrochemical impedance spectroscopy (GEIS) testing was performed on a PARSTAT electrochemical workstation by setting a loop program to perform impedance testing once every hour during a galvanostatic charge-discharge process at 0.1 C. The obtained impedance spectrum from the testing was then analyzed using EISART software (developed by Tsinghua University) for relaxation time distribution (DRT) fitting analysis [S1].

**S1.3 Materials characterization**

Microstructure and morphology of samples were characterized by using: Scanning electron microscopy (SEM, JEOL-7100F) and Transmission electron microscopy (TEM, Titan Themis G2 300). Tunneling atomic force microscopy (AFM, Bruker) for surface roughness and conductivity of OCEs. Crystal structure analysis was performed using X-ray diffraction (XRD, PANalytical) with $2\theta$ scanning ranges from 10° to 80°. Molecular structure information was confirmed by Raman spectroscopy (HORIBA LabRAM Odyssey). Mechanical properties tested by nanoindentation technology (Bruker Hysitron TI 980). Post-cycling near-surface Li^+^ distribution in OCEs was characterized by using time-of-flight secondary ion mass spectrometry (ToF-SIMS, IONTOF M6), with a sputter rate of 2.0 nm/s on SiO_2_. Lithium metal inside the OCEs was observed by Auger Electron Spectroscopy (AES, PHI 710). *In situ* differential electrochemical mass spectrometry (DEMS) was measured using a Hiden HPR-40 system with argon as the carrier gas. *In situ* optical microscopy was operated using an optical microscope (OM, Leica).

**S1.4 DFT calculation**

All computational work in this study was conducted employing the Vienna *ab initio* simulation package (VASP) within the density functional theory (DFT) framework [S2]. We utilized projector augmented wave (PAW) pseudopotentials combined with the Perdew-Burke-Ernzerhof (PBE) generalized gradient approximation (GGA) for exchange-correlation energy [S3]. To accurately account for long-range van der Waals (vdW) interactions, the empirical dispersion-corrected DFT-D3 method was implemented [S4]. The calculations employed a plane-wave energy cutoff of 500 eV and a self-consistent field (SCF) convergence threshold of 1 × 10^-5^ eV. Structural optimizations were performed using the conjugate gradient algorithm with atomic force convergence criteria set below 0.05 eV Å^-1^. Brillouin zone integrations used a Gamma-centered 1 × 1 × 1 *k*-point mesh. The convergence threshold for the iteration in the self-consistent field (SCF) was set as × 10^-5^ eV. Geometry optimization within the conjugate gradient method was performed with forces on each atom less than 0.05 eV Å^-1^. A 1 × 1 × 1 Gamma *k*-point mesh was used for the *k*-space integration. The heterojunction model built from crystalline LATP(113) and Li(110) comprised 67 Li atoms, 2 Al atoms, 10 Ti atoms, 18 P atoms and 73 O atoms. The Li(110) slab consisted of four layers while each layer contained 15 Li atoms. The heterojunction model constructed from amorphous LATP(113) and Li(110) retained the same atomic count while the crystalline LATP(113) was first rendered disordered using AIMD method and then interfaced with the Li(110) surface.

For self-consistent calculations, the cutoff energy matched that in structural optimization, and the *k*-point density was doubled in all three dimensions. The GGA+U method was employed with an effective $U=4.0 eV$ applied to Ti ions. The density of states (DOS), electrostatic potential distribution along the $c$-axis, charge density difference, and radial distribution function (RDF) between Li atoms were extracted using vaspkit [S5]. Bader charge calculations [S6] used the same *k*-point density as structural optimization.

The amorphous LATP(113) surface was prepared using *ab initio* molecular dynamics (AIMD) simulations. First, AIMD was performed in the NVT ensemble with Nosé-Hoover thermostats at 2500 K [S7]. The trajectory spanned 5000 steps ($\Delta t=1 fs$), totaling 5 ps. The system was then quenched from 2500 K to 300 K over 1 ps ($\Delta t=1 fs$) to obtain the amorphous structure.

Li^+^ diffusion properties in both samples were investigated using climbing-image nudged elastic band (CI-NEB) calculations [S8]. Parameters matched those in structural optimization.

## S1.5 Numerical simulation method

The numerical simulation was implemented using COMSOL Multiphysics®. The simulation utilized the following interfaces:

- Level set multiphase flow interface
- Dilute species transport interface
- Electric field interface

The simulation model established a rectangular domain measuring 100 μm × 100 μm. This domain was partitioned into:

- Crystal regions
- GB regions

with an average GB edge length of approximately 12 μm. The left boundary of the model was designated as the initial deposition boundary for dendrite formation.

**Dendrite simulation**

Specifically, similar to a simplified phase field method, the author simplifies the deposition and diffusion of dendrites along the GB direction into a two-phase flow problem, where a volume fraction of $\phi=1$ indicates the presence of dendrites and a volume fraction $\phi<0.95$ indicates their absence. Below are the theoretical equations used for the level set two-phase flow simulation:

|  | $\frac{\partial\phi}{\partial t}+u\cdot▽\phi=\gamma▽\cdot\left( \epsilon_{ls}▽\phi-\phi\left( 1-\phi\right)\frac{\phi}{\left\vert\phi\right\vert} \right)$ | S2 |
| --- | --- | --- |

The key parameters in the level set method are defined as follows:

- $\phi$: Level set function (dimensionless), where $\phi=0.95$ defines the interface location.

| $\bullet$ $\phi=1$: | Lithium metal. |
| --- | --- |
| $\bullet$ $\phi<0.95$: | Electrolyte. |

- $\mathbf{u}$: Interface velocity (m s^-1^), derived from electrochemical reaction kinetics.
- $\gamma$: Reinitialization parameter (stabilizes $\phi$ as a signed distance function)
- $\epsilon_{\text{ls}}$: Interface thickness control parameter (m)

where lithium deposition velocity (Interface Motion) is defined as:

|  | $u=-\frac{i}{nF}+\mu\kappa n$ | S3 |
| --- | --- | --- |

- $\mu$: Surface energy mobility (dimensionless parameter controlling interface evolution)
- $i$: Exchange current density (A m^-2^), quantifying electrochemical reaction rate
- $\kappa$: Interface curvature ($\kappa=\nabla\cdot\mathbf{n}$).

**Electric field equation**

The conservation equation of charge can be expressed as:

|  | $▽\cdot J=Q_{j}$ | S4 |
| --- | --- | --- |

- $J$: Current density (A m^-2^)
- $Q_{j}$: Current source term (A m^-3^)

And the associated the constitutive relation (Ohm’s Law) can be written as:

|  | $J=\sigma E+J_{e}$ | S5 |
| --- | --- | --- |
|  | $E=-▽V$ | S6 |

- $\sigma$: Electronic conductivity (S m^-1^)
- $E$: Electric field (V m^-1^)
- $J_{e}$: External current density (A m^-2^)
- $V$: Electric potential (V)

|  | $▽\cdot\left( \sigma▽V \right)=-Q_{j}$ | S7 |
| --- | --- | --- |

By solving the above system of equations, the electric field equation can be satisfied.

# **S2 Supplementary Text**

**S2.1 Supplementary Note S1**

# Based on the classical theory of laser-material interaction and our previous work [S9,S10], the transient rising temperature $\Delta T$ can be theoretically calculated by the following Equation about the radial distance from the center of laser beam (r), the irradiated depth on LATP pellet (z), and processing time (t):

|  | $\Delta T\left( r,z,t \right)=\frac{I_{max}\alpha\sqrt{K}}{\sqrt{\pi}\gamma}\int_{0}^{\tau} \frac{p\left( \tau-t \right)}{\sqrt{t}\left[ 1+\frac{8Kt}{W^{2}} \right]}\exp\left[ -\frac{z^{2}}{4Kt}-\frac{r^{2}}{4Kt+0.5W^{2}} \right]dt$ | S8 |
| --- | --- | --- |

The temperature profile at the irradiation center (r = 0) can be approximated using a simplified form of Equation S9, where $I_{max}$ (W m^-2^) represents the peak laser intensity, $\alpha$ denotes the material’s optical absorption coefficient, K (m^2^ s^-1^) indicates thermal diffusivity, $\gamma$ (W K^-1^ m^-1^) stands for thermal conductivity, $\tau(s)$ corresponds to pulse duration, and W (m) characterizes the beam’s mode field diameter. In this formulation, the temporal dependence of pulse intensity $p\left( t \right)$ is assumed to remain constant during analysis. This approach enables temperature estimation across varying absorption depths within the material under pulsed laser irradiation. As illustrated in Figure S1 the absorbance of LATP pellets under a UV laser at 355 nm was measured at 0.105. The relevant physical parameters of LATP are summarized in Table S4. Based on the Gaussian distribution of laser energy, the laser beam spacing of 5 $\mu s$ was set to process the surfaces of the LATP pellets to obtain the optimal uniform energy distribution. The surface of LATP pellets were processed by progressively increasing the laser power through adjusting the laser frequency to 50, 40, and 30 kHz, the corresponding parameters about theoretically calculated instantaneous temperature is shown in Table S5.

**S2.2 Supplementary Note S2**

The pulse energy fluence ($F$) and the light intensity ($I$) can be calculated using Equations S9 and S10, respectively:

|  | $F=\frac{4P}{\pi f_{L}D_{L}^{2}}$ | S9 |
| --- | --- | --- |
|  | $I=\frac{F}{\tau_{L}}=\frac{4P}{\pi f_{L}\tau_{L}D_{L}^{2}}$ | S10 |

where $P$ denotes the actual laser power incident on the substrate surface, as measured by a laser power meter; $f_{L}$ corresponds to the laser pulse frequency; $D_{L}$ represents the radius of the laser spot, maintained constant at 20$\mu m$; and $\tau_{L}$ refers to the pulse duration, which is also held constant at 1$\mu s$.

As delineated in Table S4, for a fixed set power and pulse width, the measured output power ($P$), along with the corresponding calculated values for pulse energy density ($F$) and light intensity ($I$), exhibits a clear dependence on the pulse frequency ($f_{L}$).

# **S3 Supplementary Figures**

**Fig. S1 Absorbance spectrum of LATP pellets.** Calculated temperature versus depth of near surface samples of LATP pellets. (the details of calculation see Supplementary Note S1).

**Fig. S2 Calculated temperature versus depth profile for LATP pellets.** The absorbance of the LATP pellets was measured to be 0.105 using a 355 nm UV laser.

**Fig. S3 Quantitative porosity analysis on surface SEM images.** Statistical bar chart showing surface porosity values derived from ImageJ analysis.

**Fig. S4 Ionic conductivity analysis.** EIS of pristine LATP pellet and LATP pellets treated with different laser parameters.

**Fig. S5 Raman measurement.** Raman spectra of LATP and LATP@al.

**Fig. S6 AFM measurement.** 3D AFM image (2 $\mu m$ $\mu m$) of top surface of (A) LATP and (B) LATP@al.

**Fig. S7 Rate performance.** Rate performances at current densities from 0.1 to 1.9 mA cm^-2^.

**Fig. S8 Construction of the DFT structural model.** The structure of (A) LATP | Li and (B) LATP@al | Li.

**Fig. S9** **The DOS of the corresponding interfaces.** Magnified view of the density of states for the (A) LATP | Li and (B) LATP@al | Li interfaces.

**Fig. S10 *In situ* optical monitoring for Li | LATP | Li.** Operando optical observations for Li | LATP | Li interface during continuous Li deposition at a current density of 6 mA cm^-2^. Scale bar = 100 μm.

**Fig. S11 *In situ* optical monitoring for Li | LATP@al | Li.** Operando optical observations for Li | LATP@al | Li interface during continuous Li deposition at a current density of 6 mA cm^-2^. Scale bar = 100 μm.

**Fig. S12** **SEM images.** SEM images for (A) LATP and (B) LATP@al surface after 200 h cycled in Li symmetric cell. Scale bar = 10 μm.

**Fig. S13** **Cross-sectional microstructural and elemental analysis of cycled LATP.** Cross-sectional SEM image and corresponding AES maps of O, P, Ti, Li, Al for LATP.

**Fig. S14** **Cross-sectional microstructural and elemental analysis of cycled LATP@al.** Cross-sectional SEM image and corresponding AES maps of O, P, Ti, Li, Al for LATP@al.

**Fig. S15** **EELS analysis.** The line scan regions of the EELS spectrum were marked on (**A**) LATP-based LCO and (**B**) LATP@al-based LCO, respectively.

**Fig. S16** **The charge and discharge profiles.** The voltage profiles during the first two charge and discharge cycles of Li | LATP | LCO, with interruptions for impedance spectroscopy measurements.

**Fig. S17** **The charge and discharge profiles.** The voltage profiles during the first two charge and discharge cycles of Li | LATP@al | LCO, with interruptions for impedance spectroscopy measurements.

**Fig. S18** **Interpretation of DRT analysis for Li | Ses | LCO EIS spectra.** (**A**) Representative EIS spectrum and fitting results for the Li | LATP@al | LCO cell. (**B**) Equivalent circuit model for the Li | SEs | LCO cell.

**Fig. S19** **Representative examples of DRT deconvolution results.**

**Fig. S20** ***In situ* DRT results.** *In situ* DRT results for Li | LATP | LCO during the initial two galvanostatic cycles.

**Fig. S21** ***In situ* DRT results.** *In situ* DRT results for Li | LATP@al | LCO during the initial two galvanostatic cycles.

**Fig. S22 DRT evolution contrast.** Representative profiles at equidistant intervals during the initial two cycles for (**A**) LATP@al vs. (**B**) LATP.

**Fig. S23 Full-cell performance.** The rate performance of Li | LATP | LCO and Li | LATP@al | LCO cells at 0.1, 0.2, 0.5, 1 and 2 C, respectively.

**Fig. S24** **Nail penetration test.** (**A**) Nail penetration thermal response: solid-state (LCO | LATP@al | Li) vs. liquid, (**B**) and corresponding images.

**Fig. S25** XRD patterns of LLZO and LLTO before and after laser treatment. (**A**) XRD patterns of LLZO and LLZO@al. (**B**) XRD patterns of LLTO and LLTO@al.

**Fig. S26** SEM images of LLZO and LLTO before and after laser treatment. (**A**) SEM images of LLZO and LLZO@al. (**B**) SEM images of LLTO and LLTO@al.

**Fig. S27 Interface stability assessment in LLZO system.** Durability comparison of LLZO and LLZO@al under Li symmetric cell configuration.

**Fig. S28 Interface stability assessment in LLTO system.** Durability comparison of LLTO and LLTO@al under Li symmetric cell configuration.

**Fig. S29** **Full-cell performance.** The cycling performance of Li | LLZO | LCO and Li | LLZO@al | LCO cells at 0.2 C.

**Fig. S30** **Full-cell performance.** The cycling performance of Li | LLTO | LCO and Li | LLTO@al | LCO cells at 0.2 C.

# **S4 Supplementary Tables**

**Table S1** **Dependence of UV laser output power on pulse repetition frequency**

| **Set value** | | | **Measured value** | **Calculated value** |
| --- | --- | --- | --- | --- |
| **Pulse repetition frequency**  (kHz) | **Q-pulse width**  (μs) | **Laser speed**  (mm s^-1^) | **Laser output power**  (W) | **Peak laser intensity**  (W m^-2^) |
| 50 | 1 | 300 | 1.58 | 1.00585924 × 10^11^ |
| 40 | 1 | 300 | 1.71 | 1.36077476 × 10^11^ |
| 30 | 1 | 300 | 1.82 | 1.93107998 × 10^11^ |

**Table S2** **Pulse energy density and light intensity under different laser operating conditions**

| **Set value** | **Measured value** | **Calculated value** | |
| --- | --- | --- | --- |
| **Laser frequency**  (kHz) | **Output power**  (W) | **Pulse energy density**  (J cm^-2^) | **Light intensity**  (W cm^-2^) |
| 50 | 1.58 | 10.06 | 1.00 |
| 40 | 1.71 | 13.61 | 1.36 |
| 30 | 1.82 | 19.31 | 1.93 |

**Table S3** **Summary of the cycle stability and critical current density of Li symmetric cells reported in this work and representative literature, including the main test conditions**

| **System** | **Current density**  (mA cm^-2^) | **Areal capacity**  (mAh cm^-2^) | **Stable cycling time**  (h) | **Critical current density**  (mA cm^-2^) | **Ref.** |
| --- | --- | --- | --- | --- | --- |
| Li \| PVC:BN-LATP \| Li | 0.2 | 0.1 | 800 | 1.4 | [S11] |
| Li \| CuF_2_@LATP \| Li | 0.2 | 0.2 | 2200 | 1.7 | [S12] |
| Li \| SE@KANF-LATP \| Li | 0.2 | 0.2 | 2000 | 1.4 | [S13] |
| Li \| LATP-MoS_2_@SP \| Li | 0.1 | \ | 1200 | 0.63 | [S14] |
| Li \| BN \| LATP \| BN \| Li | 0.3 | \ | 500 | \ | [S15] |
| Li \| GPE-PVDF@LATP \| Li | 0.2 | 0.2 | 600 | \ | [S16] |
| Li \| MLATP_20% \| Li | 0.2 | 0.1 | 1000 | 0.76 | [S17] |
| Li \| LATMP-DLSE \| Li | 0.1 | \ | 700 | 1.6 | [S18] |
| Li \| LATP-1.0 \| Li | 0.5 | 0.25 | 380 | 1.25 | [S19] |
| Li \| SF-B6-LATP-SF \| Li | 0.05 | \ | 2100 | 2 | [S20] |
| Li \| LATP@al \| Li | 0.2 | 0.2 | 2100 | 2.4 | This work |

**Table S4** **Comparison of electrochemical performance of reported optimization strategies for LATP-based electrolytes in SSLMBs paired with high-voltage cathodes**

| **Cathode/Anode** | **Electrolyte** | **Cathode loading**  (mg cm^-2^) | | **Voltage window** | **Stability** | **Ref.** |
| --- | --- | --- | --- | --- | --- | --- |
| LCO/Li | GCMP-LATP | ~2.5 | 3.0-4.6 V | | 50 cycles, 164.6 mAh g^-1^ capacity retention at 0.2 C (1 C=200 mAh g^-1^) | [S21] |
| LCO/Li | 1 wt% B_2_O_3_ in LATP | ~33 | 3.0-4.4 V | | 50 cycles, 66.74 mAh g^-1^ capacity retention at 0.05 C (1 C=140 mAh g^-1^) | [S22] |
| LCO/Li | LATP infiltrated with PEO | \ | 3.0-4.2 V | | 10 cycles, 105 mAh g^-1^ capacity retention at 0.05 C | [S23] |
| LCO/Li | InCI_3_@LATP-F | \ | 2.7-4.2 V | | 100 cycles, 126.4 mAh g^-1^ capacity retention at 0.5 C | [S24] |
| LCO/LiIn | LATP (CoTiO_3_) | \ | 1.7-3.7 V | | 160 cycles, 135.78 mAh g^-1^ capacity retention at 0.1 C | [S25] |
| NCM/Li | LATP/BN | ~4 | 2.5-4.25 V | | 100 cycles, 132 mAh g^-1^ capacity retention at 0.5 C (1 C=150 mAh g^-1^) | [S15] |
| NCM811/Li | SnCl_4_@LATP | 1.2-1.5 | 2.8-4.3 V | | 100 cycles, 131.94 mAh g^-1^ capacity retention at 0.5 C (1 C=180 mAh g^-1^) | [S26] |
| NCM/Li | CuF_2_@LATP | \ | 2.8-4.3 V | | 100 cycles, 98 mAh g^-1^ capacity retention at 0.5 C (1 C=180 mAh g^-1^) | [S12] |
| LCO/Li | LATP@SPE-Al | \ | 3.0-4.2 V | | 100 cycles, 120 mAh g^-1^ capacity retention at 0.5 C (1 C=170 mAh g^-1^) | [S27] |
| LCO/Li | F-LATP-LE | 2.5-3 | 3.0-4.5 V | | 300 cycles, 130.97 mAh g^-1^ capacity retention at 0.5 C | [S28] |
| LCO/Li | LATP@al | ~3.0 | 3.0-4.5 V | | 800 cycles, 101.9 mAh g^-1^ capacity retention at 0.2 C (1 C=178 mAh g^-1^) | This work |

**Table S5** **Relevant physical parameters of LATP materials**

| **Thermal diffusivity**  K (m^2^ s^-1^) | **Thermal conductivity**  $\gamma$ (W K^-1^ m^-1^) | **Light absorptivity**  α (at 355 nm) |
| --- | --- | --- |
| 8.33 × 10^-7^ | 2 | 0.105 |

# **S5 Captions for Videos**

## Video S1 Video of the process for surface amorphous LATP formation. The process of laser ablation forming an amorphous layer on LATP surface.

## Video S2 Video of the simulation process for the formation of amorphous LATP. Preparation process of simulated amorphous LATP(113) structure, including Isothermal NVT relaxation and annealing.

## Video S3 Video of phase-field simulations for the growth behavior of lithium dendrites. Phase-field modeling of lithium dendrite spatiotemporal evolution and interfacial polarization during galvanostatic deposition in LATP and LATP@al.

## Video S4 Video of time-of-flight secondary ion mass spectrometry (ToF-SIMS) analysis of lithium metal clusters. The variation in Li^+^ distribution at different depths within LATP and LATP@al.

## Video S5 Video of nail penetration tests. Nail penetration tests for Li | LATP | LCO pouch cell and Li | LATP@al | LCO pouch cell.

# **Supplementary References**

[S1] H. Li, Z. Lyu, M. Han, Robust and fast estimation of equivalent circuit model from noisy electrochemical impedance spectra. Electrochim. Acta **422**, 140474 (2022). <https://doi.org/10.1016/j.electacta.2022.140474>

[S2] J. Hafner, Ab-initio simulations of materials using VASP: Density-functional theory and beyond. J. Comput. Chem. **29**, 2044 (2008). <https://doi.org/10.1002/jcc.21057>

[S3] J. P. Perdew, W. Yang, K. Burke, Z. Yang, E. K. U. Gross, M. Scheffler, G. E. Scuseria et al., Understanding band gaps of solids in generalized Kohn–Sham theory. Natl. Acad. Sci. **114**(11), 2801-2806 (2017). <https://doi.org/10.1073/pnas.1621352114>

[S4] S. Grimme, J. Antony, S. Ehrlich, H. Krieg, A consistent and accurate ab initio parametrization of density functional dispersion correction (DFT-D) for the 94 elements H-Pu. J. Chem. Phys. **132**, 154104 (2010). <https://doi.org/10.1063/1.3382344>

[S5] V. Wang, N. Xu, J.-C. Liu, G. Tang, W.-T. Geng, VASPKIT: A user-friendly interface facilitating high-throughput computing and analysis using VASP code. Comput. Phys. Commun. **267**, 108033 (2021). <https://doi.org/10.1016/j.cpc.2021.108033>

[S6] E. Sanville, S. D. Kenny, R. Smith, G. Henkelman, Improved grid-based algorithm for Bader charge allocation. J. Comput. Chem. **28**, 899 (2007). <https://doi.org/10.1002/jcc.20575>

[S7] S. Nosé, Prog. Constant temperature molecular dynamics methods. A climbing image nudged elastic band method for finding saddle points and minimum energy paths. Theor. Phys. Suppl. **103**, 1-46 (1991). <https://doi.org/10.1143/PTPS.103.1>

[S8] G. Henkelman, B. P. Uberuaga, H. Jónsson, A climbing image nudged elastic band method for finding saddle points and minimum energy paths. J. Chem. Phys. **113**, 9901-9904 (2000). <https://doi.org/10.1063/1.1329672>

[S9] R. Yang, Y. Chen, Y. Pan, M. Kim, H. Liu et al., Single-step laser-printed integrated sulfur cathode toward high-performance lithium–sulfur batteries. Nat. Commun. **16**, 2386 (2025). <https://doi.org/10.1038/s41467-025-57755-0>

[S10] Y. Chen, M. Tan, R. Yang, C. K. W. Lee, H. Zhong et al., Laser-guided self-assembly of thin films into micro-rolls. Adv. Funct. Mater. **34**(19), 2400090 (2024). <https://doi.org/10.1002/adfm.202400090>

[S11] J. Zhang, G. Liu, P. Zhai, X. Guo, Stabilizing the interface of Li_1.3_Al_0.3_Ti_1.7_(PO_4_)_3_ and lithium electrodes via an interlayer strategy in solid-state batteries. Chem. Commun. **61**(14), 2949 (2025). <https://doi.org/10.1039/d4cc06375a>

[S12] D. Ding, H. Ma, H. Tao, X. Yang, L.-Z. Fan, Stabilizing a Li_1.3_Al_0.3_Ti_1.7_(PO_4_)_3_/Li metal anode interface in solid-state batteries with a LiF/Cu-rich multifunctional interlayer. Chem. Sci. **15**(10), 3731 (2024). <https://doi.org/10.1039/d3sc06347j>

[S13] W. Kong, Z. Jiang, Y. Liu, Q. Han, L.-X. Ding et al., Stabilizing Li_1.3_Al_0.3_Ti_1.7_(PO_4_)_3_|Li metal anode interface in solid-state batteries by Kevlar Aramid nanofiber-based protective coating. Adv. Funct. Mater. **33**(50), 2306748 (2023). <https://doi.org/10.1002/adfm.202306748>

[S14] Q. Xia, S. Yuan, Q. Zhang, C. Huang, J. Liu, H. Jin, Designing the interface layer of solid electrolytes for all-solid-state lithium batteries. Adv. Sci. **11**, 2401453 (2024). <https://doi.org/10.1002/advs.202401453>

[S15] Q. Cheng, A. Li, N. Li, S. Li, A. Zangiabadi et al., Stabilizing solid electrolyte-anode interface in Li-metal batteries by boron nitride-based nanocomposite coating. Joule **3**, 1510-1522 (2019). <https://doi.org/10.1016/j.joule.2019.03.022>

[S16] D. Yang, Y. Yang, Y. Sun, T. Zhang, A Facile Humidity-induced porous framework filled with in situ initiated gel electrolyte for stabilizing Li_1.3_Al_0.3_Ti_1.7_(PO_4_)_3_/Li interface. Adv. Funct. Mater. **35**(17), 2420202 (2025). <https://doi.org/10.1002/adfm.202420202>

[S17] C. Huang, S. Huang, A. Wang, Z. Liu, D. Pei, J. Hong et al., Stabilizing the Li_1.4_Al_0.6_Ti_1.6_(PO_4_)_3_ Li_1.3_Al_0.3_Ti_1.7_(PO_4_)_3_/Li interface with an in situ constructed multifunctional interlayer for high energy density batteries. J. Mater. Chem. A **10**(48), 25501 (2022). <https://doi.org/10.1039/d2ta07783c>

[S18] L. Song, C. Yang, L. Jiang, Y. Xiong, X. Qin et al., Structural design and performance research of high-temperature double-layer composite solid electrolyte. J. Energy Storage **143**, 119677 (2026). <https://doi.org/10.1016/j.est.2025.119677>

[S19] M. Kim, Y. W. Nahm, J. Y. Kim, K. M. Lee, Y. C. Kang, Spray pyrolysis synthesis of Li_2_O–V_2_O_5_–B_2_O_3_ glass for the low-temperature sintering of LATP electrolytes in solid-state lithium metal batteries. Small **22**, e09553 (2026). <https://doi.org/10.1002/smll.202509553>

[S20] F. Wang, M. Zhang, Z. Fang, H. Zhou, J. Wu et al., Mechano-electrical buffer layer at grain boundary induced solid state electrolyte with ultra-high mechanical strength and electrical insulation for stable lithium metal batteries. Energy Storage Mater. **77**, 104198 (2025). <https://doi.org/10.1016/j.ensm.2025.104198>

[S21] X. Yao, S. Chen, C. Wang, T. Chen, J. Li et al., Interface welding via thermal pulse sintering to enable 4.6 V solid-state batteries. Adv. Energy Mater. **14**(10), 2303422 (2024). <https://doi.org/10.1002/aenm.202303422>

[S22] X. Han, S. Wang, Y. Xu, G. Zhong, Y. Zhou et al., All solid thick oxide cathodes based on low temperature sintering for high energy solid batteries. Energy Environ. Sci. **14**(9), 5044 (2021). <https://doi.org/10.1039/d1ee01494c>

[S23] H. W. Kim, J. Kim, D. Kim, Y. Kim, W.-G. Lee, A flexible and scalable Li-ion conducting film using a sacrificial template for high-voltage all-solid-state batteries. J. Mater. Chem. A **11**(27), 14655-14662 (2023). <https://doi.org/10.1039/d3ta01194a>

[S24] W. Xiao, J. Li, C. Miao, Y. Xin, S. Nie et al., Engineering and regulating the interfacial stability between Li_1.3_Al_0.3_Ti_1.7_(PO_4_)_3_-based solid electrolytes and lithium metal anodes for solid-state lithium batteries. Colloid Interface Sci. **652**, 1447 (2023). <https://doi.org/10.1016/j.jcis.2023.08.180>

[S25] S.-C. Li, J.-G. Yu, A well-designed CoTiO_3_ coating for uncovering and manipulating interfacial compatibility between LiCoO_2_ and Li_1.3_Al_0.3_Ti_1.7_(PO_4_)_3_ in high temperature zone. Appl. Surf. Sci. **526**, 146601 (2020). <https://doi.org/10.1016/j.apsusc.2020.146601>

[S26] D. Ding, H. Tao, X. Fan, X. Yang, L.-Z. Fan, A hybrid LiCl/Li_x_Sn conductive interlayer to unlock the potential of solid-state lithium metal batteries. Adv. Funct. Mater. **34**(29), 2401457 (2024). <https://doi.org/10.1002/adfm.202401457>

[S27] L. Luo, Z. Sun, H. Gao, C. Lan, X. Huang et al., Insights into the enhanced interfacial stability enabled by electronic conductor layers in solid-state Li batteries. Adv. Energy Mater. **13**(10), 2203517 (2023). <https://doi.org/10.1002/aenm.202203517>

[S28] X.-A. Li, Y. Xu, K. Zhu, Y. Wang, Z. Zhao et al., Passerini, and Z. Chen, A facile construction of LiF interlayer and F-doping via PECVD for LATP-based hybrid electrolytes: Enhanced Li-ion transport kinetics and superior lithium metal compatibility. Mater. Today **91**, 1 (2025). <https://doi.org/10.1016/j.mattod.2025.10.003>
